# Estimating County-Level Overdose Rates Using Opioid-Related Twitter Data: Interdisciplinary Infodemiology Study

**DOI:** 10.2196/42162

**Published:** 2023-01-25

**Authors:** Raphael Cuomo, Vidya Purushothaman, Alec J Calac, Tiana McMann, Zhuoran Li, Tim Mackey

**Affiliations:** 1 School of Medicine University of California, San Diego La Jolla, CA United States; 2 Global Health Policy and Data Institute San Diego, CA United States; 3 San Diego Supercomputer Center San Diego, CA United States; 4 Department of Anthropology University of California, San Diego La Jolla, CA United States; 5 S-3 Research San Diego, CA United States

**Keywords:** overdose, mortality, geospatial analysis, social media, drug overuse, substance use, social media data, mortality estimates, real-time data, public health data, demographic variables, county-level

## Abstract

**Background:**

There were an estimated 100,306 drug overdose deaths between April 2020 and April 2021, a three-quarter increase from the prior 12-month period. There is an approximate 6-month reporting lag for provisional counts of drug overdose deaths from the National Vital Statistics System, and the highest level of geospatial resolution is at the state level. By contrast, public social media data are available close to real-time and are often accessible with precise coordinates.

**Objective:**

The purpose of this study is to assess whether county-level overdose mortality burden could be estimated using opioid-related Twitter data.

**Methods:**

International Classification of Diseases (ICD) codes for poisoning or exposure to overdose at the county level were obtained from CDC WONDER. Demographics were collected from the American Community Survey. The Twitter Application Programming Interface was used to obtain tweets that contained any of the 36 terms with drug names. An unsupervised classification approach was used for clustering tweets. Population-normalized variables and polynomial population-normalized variables were produced. Furthermore, *z* scores of the Getis Ord Gi clustering statistic were produced, and both these scores and their polynomial counterparts were explored in regression modeling of county-level overdose mortality burden. A series of linear regression models were used for predictive modeling to explore the interpretability of the analytical output.

**Results:**

Modeling overdose mortality with normalized demographic variables alone explained only 7.4% of the variability in county-level overdose mortality, whereas this was approximately doubled by the use of specific demographic and Twitter data covariates based on a backward selection approach. The highest adjusted *R*^2^ and lowest AIC (Akaike Info Criterion) were obtained for the model with normalized demographic variables, normalized *z* scores from geospatial analyses, and normalized topic counts (adjusted *R*^2^=0.133, AIC=8546.8). The *z* scores of the Getis Ord Gi statistic appeared to have improved utility over population-normalization alone. In this model, median age, female population, and tweets about web-based drug sales were positively associated with opioid mortality. Asian race and Hispanic ethnicity were significantly negatively associated with county-level burdens of overdose mortality.

**Conclusions:**

Social media data, when transformed using certain statistical approaches, may add utility to the goal of producing closer to real-time county-level estimates of overdose mortality. Prediction of opioid-related outcomes can be advanced to inform prevention and treatment decisions. This interdisciplinary approach can facilitate evidence-based funding decisions for various substance use disorder prevention and treatment programs.

## Introduction

Overdose from substance misuse remains a serious threat to public health in the United States. Concerns relating to overdose-related mortality have risen since the World Health Organization declared COVID-19 a global pandemic on March 11, 2020, given the negative effects of the pandemic on mental health and its potential cooccurrence with substance use disorder (SUD) [1]. In the United States, it is estimated that there were 100,306 drug overdose deaths from April 2020 to April 2021, with 3 quarters due to opioid use, an increase of 35% from the prior year [2]. While overdose deaths from prescription opioids and heroin have largely leveled off and are decreasing, there has been a substantial rise in overdose deaths from the use of synthetic opioids, such as fentanyl and polysubstance use [3]. Other contributing factors include mental illness, intentional and accidental poisoning from prescription medication (eg, neuroleptics, antidepressants), and occupational exposures (eg, cholinergic agents), which can result in serious injury or death [4,5].

Evidencing the ongoing severity of the national opioid public health crisis, a retrospective, multicenter study of emergency departments in Alabama, Colorado, Connecticut, North Carolina, Massachusetts, and Rhode Island from January 2018 to December 2020 found that while there was a 14% decline in all-cause emergency department visits, there was a 10.5% increase in overdose-related visits and a 28.5% increase in opioid overdose rates [6]. What was already a growing opioid disease burden at the beginning of the pandemic was attributable to factors such as social isolation, interrupted access to prevention and treatment services, and economic hardship [7]. A disparity in impact was also prominent along racial and ethnic lines, with Black Americans experiencing the largest percent increase in overdose death rates from 2019 to 2020 and American Indians and Alaska Natives experiencing the highest overdose death rates compared to other racial and ethnic groups [8-10].

The National Poison Data System (NPDS) currently collects and monitors self-reported accidental and intentional poison exposures for use by epidemiologists, state and federal agencies, and health practitioners. However, only a small amount (<5%) of NPDS-generated alerts represent incidents of public health significance [11], as many of these alerts take the form of routine automated emails derived from minor random anomalies in data received from local Poison Centers [12]. Hence, there is a clear need for alternative, big data-driven toxicosurveillance systems that can accurately use a wider breadth of covariates, including potentially analyzing self-reported incidents by the public, in order to characterize changes in burden from an overdose, especially among localities with marginalized populations [13,14]. Furthermore, examining data from a geospatial perspective has the potential to elucidate specific communities that are at higher risk for overdose-related burden.

Importantly, the lag time for reporting SUD burden may be decreased by using natural language processing applied to high-sample social media data in infodemiology and infoveillance approaches (ie, the science of distribution and determinants of information in an electronic medium) and using these covariates to build predictive models of extant SUD burden data [15]. Evidencing this potential, social media data is now widely used to conduct public health surveillance for a number of different human behaviors and health issues (eg, mental health, tobacco use, nutrition, infectious diseases), including substance use, misuse, and disorder [16,17]. For example, unsupervised machine learning has been used to identify tweets describing substance misuse and injection drug use associated with the 2015 HIV outbreak in Scott County, Indiana, and statistical approaches were used to fit demographic covariates to these tweets [18]. Infodemiology-driven approaches may also have the potential to characterize variations in predicted incidents of public health significance closer to real-time, including, but not limited to, identifying existing and new trends in the misuse of prescription drugs, polysubstance use, and risk of fentanyl exposure with high temporospatial resolution [15,18-20]. Conceptually, web-based conversations relating to a SUD-related topic are likely to represent temporally proximal phenomena to substance use and its consequent disease burden events, as discussions about these occurrences, are more likely to occur soon after, rather than long after, the occurrences themselves [21-23]. However, modeling the spatial distribution of overdose mortality has not been done via a multistep method incorporating objective text clustering, spatial aggregation, mathematical transformations of spatial covariates, and statistical modeling. An algorithm based upon these steps has the potential to be replicated efficiently, thereby allowing for estimates of public health burden with a reduced lag time when compared to official estimates.

With the overdose burden growing during the COVID-19 pandemic, there is a pressing need to assess the utility of novel public health surveillance approaches that can help identify individual and community-level variations in SUD burden, specifically mortality from an overdose. Here, the objective of this retrospective infodemiology study was to incorporate demographic data with geospatially tagged social media data from Twitter to conduct an experimental modeling exercise for generating predictions of county-level overdose death rates.

## Methods

To carry out the study objective, this interdisciplinary infodemiology study was conducted in five phases: (1) data collection of tweets associated with SUD-related keywords and slang terms, (2) characterization of tweet themes using unsupervised machine learning, (3) geospatial aggregation, (4) mathematical transformations of spatial patterns, and (5) statistical modeling to assess potential predictive value for overdose mortality. A visual summary of the methodology is provided in Figure S1 in Multimedia Appendix 1.

### Data Collection

Publicly available social media posts were retrospectively collected from Twitter in October 2021 using the Twitter Academic Application Programming Interface (API). The Twitter Academic API is a product track that includes access to all API v2 endpoints to help academic researchers use Twitter data. Compared to other APIs made available by Twitter, the Academic API can obtain larger volumes of posts in a retrospective query, though the output remains a subsample of posts that are randomly selected from the larger population of posts with user-defined specifications (eg, keywords and time frames). Based on prior studies that have identified and characterized self-reported SUD behavior by users on Twitter and an unclassified Drug Enforcement Administration intelligence report on drug slang code words, a group of keywords specific to opioid and other controlled substance drug names and slang terms were used for data collection (see Table 1) [24]. Tweets without geospatial information or that did not have at least county-level resolution (eg, tweets geotagged to the “United States” or “California, USA”) were removed prior to topic modeling. Specifically, 28,400 tweets with latitude and longitude coordinates were available from 1,266,479 tweets containing the keywords specified. Demographic data at the county level to compare to Twitter posts were available from the American Community Survey. Crude death rates due to drug overdose by county for the years 2017-2019 were obtained from the Underlying Cause of Death database on the US Center for Disease Control and Prevention Wonder data set [25]. The ICD-10 codes used to obtain crude death rates due to drug overdose are included in Multimedia Appendix 2.

**Table 1:** List of keywords used to obtain Twitter data used in model building.

Drug class	Drug name	Slang term
Opioid products	Morphine, Oxycodone, Vicodin, Oxymorphone, Codeine	Pain killer, Morph, Demmies, Dillies, Oxy, Miss Emma, Vikes, O bomb, Octagons, Captain Cody, Percs, Oxycet, Hibilly heroin, Oxycotton, Oxy 80s, Sizzurp, Purple drank, Blue heaven, Doors and floors, Rushbo, Waston-387
Other controlled substances	Xanax, Adderall, Ecstasy, or MDMA^a^	Xannax, Adderal, Happy pills
Synthetic opioids	Fentanyl	Goodfella, China white, Fetty, FettyShine, Murder 8, Tango, and cash

^a^MDMA: 3,4-methylenedioxy-methamphetamine.

### Unsupervised Machine Learning for Content Detection and Analysis

The Biterm Topic Model (BTM) was used for unsupervised topic modeling of the corpus of tweets generated from our data collection of specific opioid and drug-related keywords. We used BTM for clustering keyword-containing tweets and omitting irrelevant topics, as well as the backward selection approach used for model building for eliminating clusters of tweets with no statistical association to county-level variation in overdose mortality. Topics outputted by BTM are based upon the cooccurrence of words (ie, “biterms”) within a corpus of tweets and are particularly useful when exploring new topics, identifying new trends, and characterizing user-generated content in the absence of training data used for supervised machine learning approaches.

BTM is based on the Dirichlet distribution, with equivalent shape parameters and a prespecified *k* to denote the number of topics modeled. BTM has been demonstrated to generate improved coherence scores and intercluster distance for short-form texts when compared to other topic modeling approaches such as latent Dirichlet allocation, and hence was chosen for topic exploration of tweets associated with SUD behavior. BTM was set to output k=20 topic clusters, but topic saturation was reached with 10 clusters. To understand the types of content within each cluster, we reviewed the top 10 tweets within each cluster, with some clusters having word clusters common to drug-related topics and others consisting of “noise” (ie, topics likely not explicitly related to drugs or SUD though containing study keywords; see Table 2). The UMass coherence score, a logarithmic measure of co-occurring word frequency when adjusted for the frequency of 1 word in the biterm, was used to describe the cohesiveness of the topics generated in this study [26].

**Table 2:** Description and example keywords obtained from the first round Biterm Topic Model (BTM).

BTM topic number	Corpus percent	Topic description	Example keywords	Example tweet
Topic 1	4.0%	Common Spanish terms; drug related keywords	“drugs”; “Xanax”; “online”; “amidon”; “pour”	“*Devo prendere lo Xanax.”*
Topic 2	11.9%	Common Spanish terms; unclear topic	“que”; “de”; “mas”; “como”; “con”	“*no vean how to sell drugs online, es una trampa”*
Topic 3	9.1%	Terms relating to Viking National Football League team	“vikes”; “Vikings”; “season”; “captain”; “team”	“*tough choice. I‚Äôd do a Xanax-infused coq au vin. A total win-win.”*
Topic 4	8.8%	Drug and drug slang terms	“perc”; “drank”; “hydros”; “Xanax”; “sizzurp”	“*Y‚Äôall be letting ppl who can‚Äôt start their day without popping 4 Xanax, 2 percs, and pouring up a 4 of lean tell y‚Äôall what‚Äôs cool. Y‚Äôall some losers too”*
Topic 5	1.8%	Common Turkish terms	“bhi”; “ko”; “pedido”; “ka”; “hai”	“*anksiyete krizi garantili. b√∂yle kahvaltilarda yanimdan xanax‚Äôƒ± eksik etmem”*
Topic 6	4.0%	Common Indonesian terms	“di”; “sa”; “kalo”; “tapi” “juga”; “jadi”	“*Pota nakaapat na transaction ako sa shopee ah drugs talaga online shopping fak la na q pera”*
Topic 7	15.2%	Drug and alcohol-related slang terms	“drinking”; “blue”; “butler”; “cash”; “commons”	“*Monkish Monday and da üêª Bears....thnx Nella.. - Drinking a Blue Heaven On Earth by @monkishbrewing @ FireSky Hop Farm ‚Äî “*
Topic 8	7.7%	Real estate terms; Hindi terms	“floors”; “jodhpur”; “kitchen”; “use”; “walls”	“*I‚Äôm going to buy a dozen Xanax tablets the size of an Ivory soap bar and just use them starting on Tuesday morning.”*
Topic 9	32.9%	General online drug selling terms	“buy”; “drugs”; “online”; “money”; “think”	“*eddie that was caught up in the 80s selling drugs and pimpin‚Äô then started smoking a little crack &gt; white nyc gentrifiers on oxy askin”*
Topic 10	4.7%	Crime-related terms	'police', ‘murder’, 'hospital', 'blood', 'justice', 'donate'	“*Police were called to Larchmont Road, Leicester, at 8.15pm today (Saturday 26 September) after a collision involving 3 vehicles. A 32-year-old man was arrested on suspicion of drink driving and driving whilst disqualified.”*

### Geospatial, Statistical, and Predictive Model Building

Tweets from topics featuring drug-related terms (ie, topics 1, 4, 7, and 9) were used for further geospatial and statistical analyses (although modeling conducted with all 10 topics is presented in Table S1 in Multimedia Appendix 1 for comparison). Tweets corresponding to each selected topic were geolocated, aggregated to the county level, and normalized to the county-level population. The Getis Ord Gi statistic was used to calculate *z* scores for the geospatial clustering of tweets for each selected BTM topic. In simple terms, for a given value (eg, number of tweets), the Getis Ord Gi statistic determines whether a county is part of a high-value cluster (ie, hot spot) or low-value cluster (ie, cold spot) by determining whether the observed values for that county and nearby counties significantly deviate from expected values, which are based off the entire (ie, national) set of values [27]. The *z* scores produced from this statistic provide a quantifiable gradient of high-to-low value clustering for a given county in the context of neighboring counties. Polynomial terms were computed for normalized counts and normalized *z* scores of the Getis Ord Gi statistic, thereby producing 4 mathematical representations of geospatial distributions for each of the 9 BTM topic clusters. These 4 statistics were also computed for each of the following 22 demographic variables: (1) race and ethnicity: Caucasian, African American, American Indian or Alaska Native, Asian, Native Hawaiian or Pacific Islander, Hispanic, other race, multiracial; (2) sex: male or female; and (3) age: under 5 years, 5-9 years, 10-14 years, 15-19 years, 20-24 years, 25-34 years, 35-44 years, 45-54 years, 55-64 years, 65-74 years, 75-84 years, and over 85 years.

The estimated actual rates of death were missing for 44.6% (1403/3143) of US counties. In order to create an estimate of the death data for these counties, the values of neighboring spatial features were used to impute values for the counties with missing data. Using the space-time pattern mining toolbox in ArcGIS (Esri), the estimated actual rates of death due to drug overdose were imputed for counties with missing data [28]. The spatial relationships were conceptualized using the continuity edges corner option, in which neighboring counties that share a boundary, share a node, or overlap will influence the computations. The actual rates of death due to drug overdose (including imputed values for missing data) were used for further geospatial visualizations and analyses. Geospatial analysis was conducted in ArcGIS Pro 2.9.

While Twitter data included posts from 2012-2021, the estimated actual death rates due to drug overdose (including imputed data) used in the model were from 2017-2019, inclusive. Linear regression was used for predictive modeling to facilitate the interpretability of the analytical output. Specifically, a series of initial models that included normalized demographic variables, normalized BTM topic counts, and normalized *z* scores from geospatial analyses were followed by polynomial normalized demographic variables, polynomial normalized BTM topic counts, and polynomial normalized *z* scores from geospatial analyses that were used separately and in combination to select final models with a minimum prediction criterion that could significantly predict the spatial distribution of mortality rates from a drug overdose. Normalized *z* scores and counts for all topics were made available to a backward selection algorithm in order to enable higher prediction and also to illustrate the generation of models using an automated approach. The function step Akaike information criterion (AIC) in the MASS library on R was used to select at each step the model that minimizes the prediction criterion AIC [29]. The adjusted *R*^2^ and AIC values for each of the final models were calculated. The effect estimates and model statistics were computed for the selected models with the highest adjusted *R*^2^ values. Using the model with the highest adjusted *R*^2^, the predicted rates of death due to drug overdose were calculated for each county. Statistical analyses were performed using R (version 3.6.1; R Foundation for Statistical Computing).

The ratio of predicted to actual rates of death due to drug overdose was calculated for each county using the model with the highest R-squared produced by this study (see Table S2 in Multimedia Appendix 1), to provide an illustration of predictive power using the full breadth of geospatial and statistical covariates generated with the techniques discussed in this study. Descriptive statistics were computed for the actual and predicted rates of death due to drug overdose as well as the ratio between the two. Heatmaps were created for (1) actual rates of death due to drug overdose, (2) predicted rates of death due to drug overdose, and (3) the ratio of predicted to actual rates. The heatmaps were color-coded based on the natural breaks algorithm for actual rates of death due to drug overdose, and the same intervals were used for the heatmap on predicted rates of death due to drug overdose. The average ratio of predicted to actual rates was calculated for each state based on county-level data. Heatmaps were created using ArcGIS Desktop 10.7.1.

### Ethical Considerations

As this study did not involve people, medical records, human tissues, or any other personally identifiable information, institutional review board approval was not required.

## Results

There were 28,400 geospatially identifiable Twitter posts containing the designated keywords, published between December 2012 and September 2021. This corpus was divided into 10 topics using the BTM algorithm. The overall UMass coherence score was –1505, within a typical range for coherence for topic modeling of short-form texts. The BTM coherence score was –1281 for topic 1, –1318 for topic 2, –1447 for topic 3, –1643 for topic 4, –1599 for topic 5, –1389 for topic 6, –1722 for topic 7, –1618 for topic 8, –1593 for topic 9, and –1505 for topic 10; indicating scores consistent with those for high-term corpuses in seminal work used to relay the cohesiveness of output from BTM [30]. Topics 1, 4, 7, and 9 were chosen for further modeling as their themes conveyed associations with drug-related activity. Mathematical transformations of spatial patterns were used to generate 14 initial models (see Table 3). Based on the stepwise AIC method, the highest adjusted *R*^2^ and lowest AIC were obtained for the model with normalized demographic variables, normalized *z* scores from geospatial analyses, and normalized topic counts (adjusted *R*^2^=0.133, AIC=8546.8), followed by the model with normalized demographic variables and normalized *z* scores from geospatial analyses (adjusted *R*^2^=0.131, AIC=8548.6). In the final model with the highest adjusted *R*^2^, the transformation of Twitter data covariates to *z* scores of the clustering statistic was common, except for topic 4, which was represented as a count (see Table 4). The difference in the adjusted *R*^2^ between the model with demographic variables, geospatial *z* scores, and topic counts and the model with only demographic variables was 0.059. Hence, an additional 5.9% variability in the rate of death due to drug overdose was able to be predicted by using normalized geospatial *z* scores of SUD-related Twitter posts, when compared to a model with demographic covariates alone.

**Figure 1:**
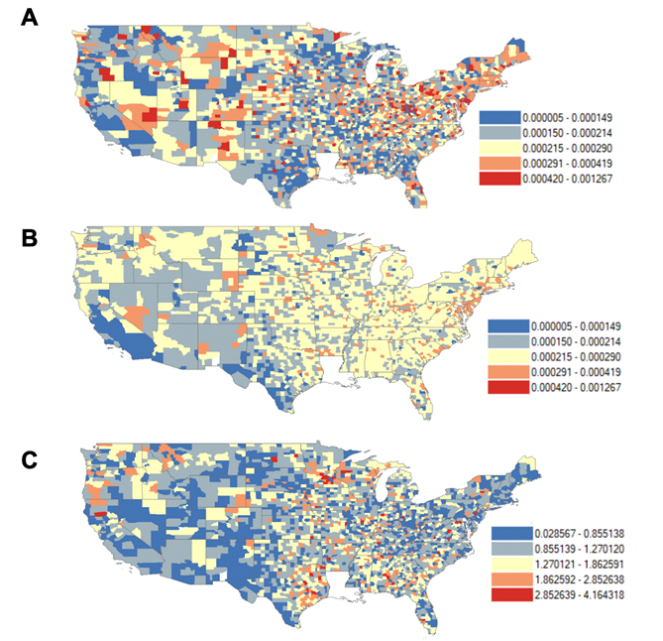
Rates of death due to drug overdose by county, United States. (A) Actual rates of death due to drug overdose (2017-2019; imputed values for counties with missing data); (B) Predicted rates of death due to drug overdose using geocoded Twitter data; (C) Ratio of predicted to actual rates of death due to drug overdose.

The average actual rate of death due to drug overdose (21.62 per 100,000) was comparable with the average predicted rate of death due to drug overdose (22.00 per 100,000). In addition, the average ratio of predicted overdose to actual overdose was 1.14. Carver County in Minnesota had the highest ratio of predicted to actual rates (4.16). While the highest actual rate of death due to drug overdose was in Cabell County in West Virginia (126.73 per 100,000), the highest predicted rate of death due to drug overdose was in Queen Anne's County in Maryland (37 per 100,000), indicating a regression toward the mean for modeled output. The highest difference of 25.3 per 100,000 between predicted and actual rates was observed in Charlotte County, Florida. The actual and predicted rates of death due to drug overdose, and the ratio between the two for each county, are visualized in Figure 1. At the state level, Alabama had the highest average ratio of predicted to actual rates of death due to drug overdose (1.58) and the highest average difference between predicted and actual rates of death due to drug overdose (5.94 per 100,000). The average predicted rate of death due to drug overdose fell within 20% accuracy for 15 states: Connecticut, Delaware, Hawaii, Illinois, Kansas, New Hampshire, New Mexico, New York, North Dakota, Oklahoma, Rhode Island, Utah, Vermont, Virginia, and Wyoming.

A model with normalized demographic covariates alone explained only 7.4% of the spatial variability in overdose mortality, which was not improved by the use of polynomial terms. However, the model with the highest coefficient of determination did include several demographic terms. These included 2 racial covariates, suggesting that modeling spatial patterns for overdose mortality would optimally take into account race-based disparities at the community level. Specifically, in this final model, Asian race and Hispanic ethnicity were significantly negatively associated with county-level burdens of overdose mortality, indicating that areas with a relatively greater burden of overdose mortality do not include high concentrations of these racial and ethnic groups.

**Table 3:** Model prediction of drug overdose death rates using stepwise Akaike information criterion (AIC).

Number	Initial model	Final model variables	Model AIC	Model adjusted *R*^2^	Model *P* value
1	Normalized demographic variables	Median age Female population Asian population Hispanic population	8658.0	0.074	<.001
2	Normalized geospatial z scores (topics 1, 4, 7, and 9)	Topic 1 z scores Topic 4 z scores Topic 9 z scores	8706.0	0.048	<.001
3	Normalized BTM topic counts (topics 1, 4, 7, and 9)	Topic 7 count Topic 9 count	8787.2	0.001	.12
4	Normalized demographic variables Normalized geospatial z scores (topics 1, 4, 7, and 9)	Median age Female population Asian population Hispanic population Topic 1 z scores Topic 9 z scores	8548.6	0.131	<.001
5	Normalized demographic variables Normalized BTM topic counts (topics 1, 4, 7, and 9)	Median age Female population Asian population Hispanic population Topic 1 count Topic 9 count	8651.1	0.079	<.001
6	Normalized geospatial z scores Normalized BTM topic counts (topics 1, 4, 7, and 9)	Topic 1 z scores Topic 4 z scores Topic 7 z scores Topic 9 z scores Topic 1 count	8705.1	0.049	<.001
7	Normalized demographic variables Normalized geospatial z scores Normalized BTM topic counts (topics 1, 4, 7, and 9)	Median age Female population Asian population Hispanic population Topic 1 z scores Topic 4 z scores Topic 9 z scores Topic 4 count	8546.8	0.133	<.001
8	Polynomial normalized demographic variables	Median age Female population Male population Hispanic population Black population White population American Indian population Other race population	8668.2	0.071	<.001
9	Polynomial normalized geospatial z scores (topics 1, 4, 7, and 9)	Topic 1 z scores Topic 4 z scores Topic 9 z scores	8739.1	0.029	<.001
10	Polynomial normalized BTM topic counts (topics 1, 4, 7, and 9)	Topic 1 count Topic 9 count	8784.9	0.003	.04
11	Polynomial normalized demographic variables Polynomial normalized geospatial z scores (topics 1, 4, 7, and 9)	Median age Female population Male population Hispanic population Black population White population American Indian population Native Hawaiian or Pacific Islander population Other race population Multiple race population Topic 1 z scores Topic 9 z scores	8596.2	0.110	<.001
12	Polynomial normalized demographic variables Polynomial normalized BTM topic counts (topics 1, 4, 7, and 9)	Median age Female population Male population Hispanic population Black population White population American Indian population Other race population Topic 1 count Topic 9 count	8662.4	0.075	<.001
13	Polynomial normalized geospatial z scores Polynomial normalized BTM topic counts (topics 1, 4, 7, and 9)	Topic 1 z scores Topic 4 z scores Topic 9 z scores Topic 9 count	8737.2	0.031	<.001
14	Polynomial normalized demographic variables Polynomial normalized geospatial z scores Polynomial normalized BTM topic counts (topics 1, 4, 7, and 9)	Median age Female population Male population Hispanic population Black population White population American Indian population Native Hawaiian or Pacific Islander population Other race population Multiple race population Topic 1 z scores Topic 9 z scores Topic 9 count	8593.7	0.112	<.001

**Table 4:** Model prediction of drug overdose death rates using normalized demographic variables, normalized z scores from geospatial analyses, and normalized Biterm Topic Model topic counts (topics 1, 4, 7, and 9; model adjusted *R*^2^=0.133, Akaike information criterion [AIC]=8546.8).

Coefficients	Estimate	SE	*P* value
Intercept	–18.39	9.26	.047
Median age	0.57	0.07	<.001
Female population	38.96	17.73	.03
Asian population	–49.75	10.01	<.001
Hispanic population	–8.73	2.60	<.001
Topic 1 *z* scores	–3.63	0.48	<.001
Topic 4 *z* scores	–0.43	0.27	.11
Topic 9 *z* scores	3.39	0.33	<.001
Topic 4 count	4.19×10^4^	2.04×10^4^	.04

## Discussion

### Principal Findings

This study computed models for rates of overdose mortality by incorporating mathematically transformed spatial distributions based on geotagged social media posts from Twitter with SUD-related keywords. In our final model, the average predicted county-level overdose mortality was similar to the actual county-level rate of overdose mortality, 23.29 per 100,000 residents and 22.00 per 100,000 residents, respectively, and the average ratio of predicted to actual mortality was 1.22 (compared to 1.14 for modeling with the full range of topics; Table S2 in Multimedia Appendix 1). At the state level, the average predicted to actual mortality ratio for 26 states fell within 20% accuracy, with a range between 0.78 and 1.58. The *z* scores of the Getis Ord Gi statistic appeared to have improved utility over population-normalization alone. In this model, median age, female population, and tweets about web-based drug sales were positively associated with opioid mortality. Asian race and Hispanic ethnicity were significantly negatively associated with county-level burdens of overdose mortality.

Regression-based model-fitting enables the generation of beta coefficients such that prediction follows predetermined patterns using a priori inputs and therefore may be preferable to epidemiologists when compared to black-box approaches such as neural networks or ensemble methods. This is to say that the output generated by this approach enables a set of disease burden estimates, and therefore, despite threats to accuracy, this approach permits the generation of a baseline for which increases and decreases can be recorded, especially from social media covariates that can be updated in frequent temporal cross-sections. Previous studies have used similar techniques to predict opioid-related outcomes, including the use of demographic variables and medications dispensed to predict 2-year overdose risk for individuals on chronic opioid therapy [31], multivariate regression modeling to predict opioid-induced respiratory depression using clinical characteristics [31], and modeling unintentional drug overdose using law enforcement drug seizure data [32]. Additionally, past studies have used similar methodologies to leverage novel data for the prediction of various other public health outcomes, such as the use of lifestyle and metabolic covariates on global pancreatic cancer incidence and mortality [33], smoking’s effect on patient-reported outcomes following orthopedic surgery [34], and alcohol and hypertension’s effect on kidney cancer incidence and mortality [35]. This study’s methodology builds upon past research by adding Twitter, a popular social media data source, alongside readily available demographic data to demonstrate how these approaches could have utility in estimating variations in overdose and SUD burden.

Generally, results demonstrate the potential benefit of using social media data as a supplement to demographic data for enabling earlier detection of overdose mortality, potentially as granular as the census tract level, which includes the benefit of a boost to the goodness of fit. Specifically, we observed that an approach involving social media data, geospatial statistics, and mathematical transformations produced about double the model coefficient of determination when compared to an approach without these data and methods. Though a backward selection approach using user-generated Twitter data covariates to model real-world public health statistics of overdose mortality may have its limitations (discussed below), there nevertheless appears to be added utility to the incorporation of these data for analyses that endeavor toward resolute, short-term prediction. The utility of this approach may also be strengthened with the integration of statistical approaches to detect aberrations, such as the Early Aberration Reporting System, though public reports suggest that these have thus far only been applied to observe, rather than modeled, data [36,37]. Further, the use of larger and more representative data sets from social media platforms (eg, data from the full Twitter firehose limited to geocoded data or other popular social media platforms that allow for geotagging) filtered for a greater number of SUD-related keywords may yield greater power and improve predictability.

During the COVID-19 pandemic, a rapid rise in opioid-related overdose deaths was observed, but reporting on this data lagged behind that of those experiencing SUD [38]. This indicates an urgent need for improved public health surveillance to ensure that interventions are more targeted and that federal, state, tribal, and local governments have sufficient data and evidence to appropriately invest in harm reduction resources [39]. Insights into which populations and communities have been most affected are crucial [40], particularly in the context of those people disproportionately impacted by *both* COVID-19 and SUD. Hence, interdisciplinary approaches such as those used in this study warrant further exploration and validation to assess their utility in generating multimodal data-driven predictions of SUD risk and burden [40]. Similarly, public health practitioners may benefit from these techniques to advance the prediction of opioid-related outcomes to inform data-driven prevention and treatment decisions targeted for specific communities that may help with SUD prevention and treatment funding. For example, despite seemingly successful statewide policy implementation, a state-by-state analysis reveals that naloxone funding remains a challenge, despite the clear benefits of harm mitigation for opioid use disorder [39].

### Limitations

Findings from this exploratory study are subject to a number of limitations. First, this modeling exercise was used with existing overdose mortality data, whereas the purported benefit is to use future rounds of social media data for closer to real-time prediction. For this reason, the utility of this study lies in the linear equation generated by the final model, which does not explain a high proportion of variability. Therefore, the value added by this study is primarily in the approach demonstrated (which can be iterated upon with more comprehensive or explanatory coefficients) rather than the linear equation itself. However, it should be noted that the authors intentionally sacrificed the predictive power of the model for the interpretability of covariate-specific beta coefficients, given the utility of a linear model for inputting future cross-sections of real-time data. Notably, we observed state-level heterogeneity in model performance, indicating that surveillance efforts leveraging a linear modeling approach should consider computing sets of beta coefficients for states separately. Data collection may have been limited due to the list of search terms used in this study, which may not be complete due to the continued addition and deletion of slang terms used among those using these drugs, though the list used in this study was based on existing literature and a Drug Enforcement Administration intelligence report. Second, the outcome variable used in this study represents 3 years of overdose deaths (2017-2019), and the Twitter data predictors were collected over 9 years (2012-2021) to enable sufficient sample sizes for modeling algorithms. Though the 2 periods intersect, the purported benefit of closer to real-time modeling is limited in that Twitter data patterns do not immediately precede outcome data. Optimal temporality was not feasible in this study due to the sample size required to detect geospatial patterns after aggregation to over 3000 county bins, which is why Twitter data predictors were derived from a lengthy period of data collection. Third, estimated actual death rates due to drug overdose were imputed for counties with missing data using space-time pattern mining tools. Imputation can lead to narrower CIs with an underestimation of standard errors and an overestimation of test statistics. Additionally, this study normalized using the total county-level population rather than the number of Twitter users, as the total number of users from a given county is not made available by Twitter, and measures of this variation from Twitter APIs are unreliable due to the limitations of API calls, which produce a corpus that falls short of the sample required to assess this variation. Finally, though the model incorporates demographic predictors, the difficulties in attributing demographic characteristics to near real-time data (ie, social media posts) represent a challenge for discrepant trends across demographic groups and, practically, for updating a priori inputs that enable socioculturally sensitive prediction.

### Conclusions

The model described in this study uses a relatively novel approach involving unsupervised topic modeling and geocoded social media posts and shows the initial feasibility of the use of infodemiology principles to generate a near-real-time prediction of overdose mortality vis a vis keyword-based Twitter activity. The results from this study, though exploratory, coupled with additional data-driven research, can facilitate evidence-based funding decisions for statewide programs that can positively impact a wide array of SUD prevention approaches, including naloxone availability to prescription drug monitoring programs.

## Appendix

Multimedia Appendix 1 Supplementary material.

Multimedia Appendix 2 List of International Classification of Diseases (ICD) Codes Associated With Overdose.

## Abbreviations

AIC: Akaike Info Criterion
API: Application Programming Interface
BTM: Biterm Topic Model
NPDS: National Poison Data System
SUD: substance use disorder

## Data Availability

The data sets generated or analyzed during this study are available from the corresponding author upon reasonable request.
