# Supplementary material for: Estimating County-Level Overdose Rates Using Opioid-Related Twitter Data: Interdisciplinary Infodemiology Study
Source: JMIR Form Res. 2023 Jan 25;7:e42162. doi: 10.2196/42162 (PMC9909516; doi:10.2196/42162)
Supplement: Multimedia Appendix 1 [file formative_v7i1e42162_app1.doc]

**Multimedia Appendix 1**

**Figure S1.** Overview of study methodology.


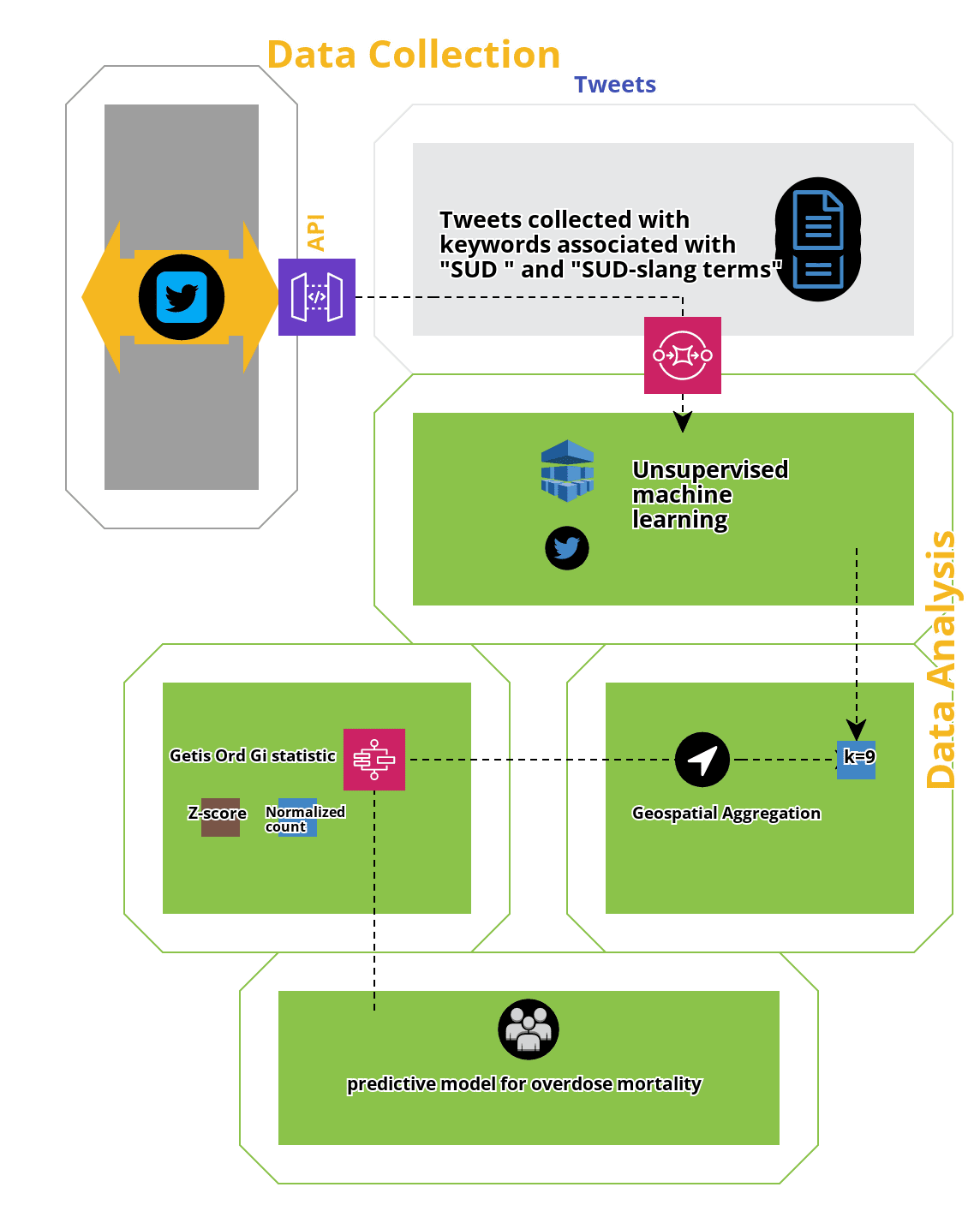


**Table S1.** Model prediction of drug overdose death rates using stepwise AIC (Akaike’s Info Criterion).

| Sl.no. | Initial Model | Final Model Variables | Model AIC | Model adjusted R^2^ | Model  *P*-value |
| --- | --- | --- | --- | --- | --- |
| 1. | Normalized demographic variables | Median age  Female population  Asian population  Hispanic population | 8658.0 | .074 | <.001 |
| 2. | Normalized geospatial Z-scores | Topic 1 Z-scores  Topic 3 Z-scores  Topic 5 Z-scores  Topic 8 Z-scores  Topic 9 Z-scores | 8697.0 | .053 | <.001 |
| 3. | Normalized BTM topic counts | Topic 2 count  Topic 9 count | 8786.8 | .002 | .095 |
| 4. | Normalized demographic variables  Normalized geospatial Z-scores | Median age  Female population  Asian population  Hispanic population  Topic 1 Z-scores  Topic 5 Z-scores  Topic 8 Z-scores  Topic 9 Z-scores | 8537.2 | .138 | <.001 |
| 5. | Normalized demographic variables  Normalized BTM topic counts | Median age  Female population  Asian population  Hispanic population  Topic 1 count  Topic 9 count | 8651.1 | .079 | <.001 |
| 6. | Normalized geospatial Z-scores  Normalized BTM topic counts | Topic 1 Z-scores  Topic 3 Z-scores  Topic 5 Z-scores  Topic 8 Z-scores  Topic 9 Z-scores  Topic 1 count  Topic 2 count | 8696.0 | .056 | <.001 |
| 7. | Normalized demographic variables  Normalized geospatial Z-scores  Normalized BTM topic counts | Median age  Female population  Asian population  Hispanic population  Topic 1 Z-scores  Topic 5 Z-scores  Topic 8 Z-scores  Topic 9 Z-scores  Topic 8 count | 8533.3 | .141 | <.001 |
| 8. | Polynomial normalized demographic variables | Median age  Female population  Male population  Hispanic population  Black population  White population  American Indian population  Other race population | 8668.2 | .071 | <.001 |
| 9. | Polynomial normalized geospatial Z-scores | Topic 1 Z-scores  Topic 3 Z-scores  Topic 5 Z-scores  Topic 8 Z-scores  Topic 9 Z-scores  Topic 10 Z-scores | 8732.0 | .035 | <.001 |
| 10. | Polynomial normalized BTM topic counts | Topic 1 count  Topic 9 count | 8784.9 | .003 | .037 |
| 11. | Polynomial normalized demographic variables  Polynomial normalized geospatial Z-scores | Median age  Female population  Male population  Hispanic population  Black population  White population  American Indian population  Native Hawaiian/Pacific Islander population  Other race population  Multiple race population  Topic 1 Z-scores  Topic 3 Z-scores  Topic 5 Z-scores  Topic 8 Z-scores  Topic 9 Z-scores  Topic 10 Z-scores | 8585.0 | .118 | <.001 |
| 12. | Polynomial normalized demographic variables  Polynomial normalized BTM topic counts | Median age  Female population  Male population  Hispanic population  Black population  White population  American Indian population  Other race population  Topic 1 count  Topic 9 count | 8662.4 | 0.075 | <0.001 |
| 13. | Polynomial normalized geospatial Z-scores  Polynomial normalized BTM topic counts | Topic 1 Z-scores  Topic 3 Z-scores  Topic 5 Z-scores  Topic 8 Z-scores  Topic 9 Z-scores  Topic 10 Z-scores  Topic 2 count  Topic 3 count  Topic 8 count | 8727.5 | 0.039 | <0.001 |
| 14. | Polynomial normalized demographic variables  Polynomial normalized geospatial Z-scores  Polynomial normalized BTM topic counts | Median age  Female population  Male population  Hispanic population  Black population  White population  American Indian population  Native Hawaiian/Pacific Islander population  Other race population  Multiple race population  Topic 1 Z-scores  Topic 3 Z-scores  Topic 5 Z-scores  Topic 8 Z-scores  Topic 9 Z-scores  Topic 10 Z-scores  Topic 8 count | 8577.6 | 0.122 | <0.001 |

**Table S2.** Model prediction of drug overdose death rates using normalized demographic variables, normalized Z-scores from geospatial analyses, and normalized BTM topic counts (Model adjusted R^2^=0.141, AIC=8533.3).

| Coefficients | Estimate | S.E. | *p*-value |
| --- | --- | --- | --- |
| Intercept | -17.42 | 9.22 | .059 |
| Median age | .58 | .07 | <.001 |
| Female population | 35.63 | 17.64 | .044 |
| Asian population | -49.16 | 9.96 | <.001 |
| Hispanic population | -8.49 | 2.58 | <.001 |
| Topic 1 Z-scores | -3.59 | .48 | <.001 |
| Topic 5 Z-scores | -1.10 | .69 | .113 |
| Topic 8 Z-scores | -1.32 | .33 | <.001 |
| Topic 9 Z-scores | 3.87 | .35 | <.001 |
| Topic 8 count | 1.35x10^5^ | .55x10^5^ | .015 |
