# Supplementary material for: Estimating County-Level Overdose Rates Using Opioid-Related Twitter Data: Interdisciplinary Infodemiology Study
Source: JMIR Form Res. 2023 Jan 25;7:e42162. doi: 10.2196/42162 (PMC9909516; doi:10.2196/42162)
Supplement: Multimedia Appendix 2 [file formative_v7i1e42162_app2.doc]

**Multimedia Appendix 2**

***List of ICD Codes Associated With Overdose***

X40: Accidental poisoning by and exposure to non-opioid analgesics, antipyretics and anti-rheumatics; X41: Accidental poisoning by and exposure to antiepileptic, sedative-hypnotic, antiparkinsonism and psychotropic drugs, not elsewhere classified; X42: Accidental poisoning by and exposure to narcotics and psychodysleptics [hallucinogens], not elsewhere classified; X43: Accidental poisoning by and exposure to other drugs acting on the autonomic nervous system; X44: Accidental poisoning by and exposure to other and unspecified drugs, medicaments and biological substances; X60: Intentional self-poisoning by and exposure to non-opioid analgesics, antipyretics and anti-rheumatics; X61: Intentional self-poisoning by and exposure to antiepileptic, sedative-hypnotic, anti-parkinsonism and psychotropic drugs, not elsewhere classified; X62: Intentional self-poisoning by and exposure to narcotics and psychodysleptics [hallucinogens], not elsewhere classified; X63: Intentional self-poisoning by and exposure to other drugs acting on the autonomic nervous system; X64: Intentional self-poisoning by and exposure to other and unspecified drugs, medicaments and biological substances; X85: Assault by drugs, medicaments and biological substances; Y10: Poisoning by and exposure to nonopioid analgesics, antipyretics and antirheumatics, undetermined intent; Y11: Poisoning by and exposure to antiepileptic, sedative-hypnotic, antiparkinsonism and psychotropic drugs, not elsewhere classified, undetermined intent; Y12: Poisoning by and exposure to narcotics and psychodysleptics [hallucinogens], not elsewhere classified, undetermined intent; Y13: Poisoning by and exposure to other drugs acting on the autonomic nervous system, undetermined intent; and Y14: Poisoning by and exposure to other and unspecified drugs, medicaments and biological substances, undetermined intent.
